# Size-dependent bending modulus of nanotubes induced by the imperfect boundary conditions

**DOI:** 10.1038/srep38974

**Published:** 2016-12-12

**Authors:** Jin Zhang

**Affiliations:** 1 Shenzhen Graduate School, Harbin Institute of Technology, Shenzhen 518055, China

## Abstract

The size-dependent bending modulus of nanotubes, which was widely observed in most existing three-point bending experiments [e.g., J. Phys. Chem. B 117, 4618–4625 (2013)], has been tacitly assumed to originate from the shear effect. In this paper, taking boron nitride nanotubes as an example, we directly measured the shear effect by molecular dynamics (MD) simulations and found that the shear effect is not the major factor responsible for the observed size-dependent bending modulus of nanotubes. To further explain the size-dependence phenomenon, we abandoned the assumption of perfect boundary conditions (BCs) utilized in the aforementioned experiments and studied the influence of the BCs on the bending modulus of nanotubes based on MD simulations. The results show that the imperfect BCs also make the bending modulus of nanotubes size-dependent. Moreover, the size-dependence phenomenon induced by the imperfect BCs is much more significant than that induced by the shear effect, which suggests that the imperfect BC is a possible physical origin that leads to the strong size-dependence of the bending modulus found in the aforementioned experiments. To capture the physics behind the MD simulation results, a beam model with the general BCs is proposed and found to fit the experimental data very well.

In the past decades, the discovery of the superior mechanical and other physical properties in quasi-one-dimensional tubular nanomaterials such as carbon nanotubes (CNTs) and boron nitride nanotubes (BNNTs) have triggered great interest in their possible engineering applications. For example, owing to their high stiffness and strength, low density and large aspect ratio, CNTs and BNNTs are proposed to be the ultimate material for the use as nanomechanical resonators for a variety of applications^1^,^2^,^3^. In addition, the extremely high elastic modulus of CNTs and BNNTs reported in the theoretical and experimental studies suggests that, compared with the conventional nanofibers, CNTs and BNNTs can be regarded as a better reinforcement for the nanocomposites^4^,^5^,^6^. Thus, to make CNTs and BNNTs be successfully employed in the aforementioned applications, a better understanding of their mechanical properties is required.

To characterize the mechanical properties of nanotubes a variety of experimental approaches have been proposed^7^,^8^, among which the three-point bending (TPB) test conducted with the atomic force microscope (AFM) is widely used^9^,^10^,^11^,^12^,^13^,^14^,^15^,^16^. In such AFM-based TPB test nanotubes are deposited onto a stiff substrate with a topographical pattern, such as polished porous aluminium oxide membranes or silica gratings patterned with trenches (see Fig. 1a). As a result, some nanotube samples can occasionally lie over pores or trenches. During the TPB test the midpoint of the suspended portion of nanotubes is subjected to a downward force applied by the AFM tip, which will induce the transverse deflection of nanotubes. Force (*F*)-displacement (*δ*) curves are recorded, and the bending modulus *E*_*b*_ thus can be calculated directly from the slope of the *F*-*δ* curve together with the second moment of area *I* and the suspended length *L* by using following equation^11^,^12^,^13^,^16^


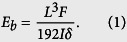


Theoretically, the bending modulus of nanotubes obtained from the TPB test is expected to be equivalent to the Young’s modulus measured by the direct tensile test. However, in contrast to the result of the tensile test^17^ a unique size-dependent bending modulus was reported in most existing TPB tests of CNTs and BNNTs^9^,^10^,^11^,^12^,^13^,^14^,^15^. To explain the size-dependent elastic modulus of nanotubes observed in the TPB tests, a transverse shear theory was proposed by Salvetat *et al*.^11^ initially in 1999 and then widely adopted by many other researchers^12^,^13^,^14^,^15^. By tacitly assuming that the size-dependence phenomenon originates from the transverse shear effect, the “curve fitting technique” was used to explain the size-dependence of the bending modulus. However, the shear modulus obtained by fitting the shear theory to the experimental results was usually found to be two orders of magnitude lower than the results measured via the torsion tests^18^ and the results calculated by the theoretical simulations^19^. This discrepancy makes us believe that a direct measurement of the transverse shear deformation is desired and some new theories may need to be formulated to reveal the physics behind the size-dependence of the bending modulus observed in the TPB tests. In addition, it is noticed that Eq. 1 is derived based on the classical Euler-Bernoulli beam model with the assumption that the ends of the beam are perfectly fixed. But in reality, we can see from Fig. 1a and b that in the TPB experiments a nanotube is usually deposited onto the surface of a substrate^9^,^10^,^11^,^12^,^13^,^14^,^15^,^16^. Under this circumstance, only a few rather than all atoms in the portion attaching to the substrate are blocked, so the ends of nanotubes may not be perfectly restricted. Therefore, the perfectly fixed ends assumed in the previous studies^9^,^10^,^11^,^12^,^13^,^14^,^15^ cannot exactly describe the real BCs of the nanotubes tested in the TPB tests. We need to abandon the assumption of the perfectly fixed ends and study the influence of BCs on the bending behaviours of nanotubes.

Motivated by these ideas, in this paper molecular dynamics (MD) simulations are performed to qualify the influence of the imperfect BCs on the bending behaviours of nanotubes. By changing the number of the blocked atoms at the ends of nanotubes, we reveal the effect of the imperfect BCs on the bending modulus and the atomic displacements of both single-walled (SW) and multi-walled (MW) nanotubes. Similar to the influence of the shear effect, when the imperfect BCs are considered the size-dependent bending modulus is also detected for nanotubes. Moreover, by comparing these two theories quantitatively, we identify the influence of the imperfect BCs as the possible major factor responsible for the strong size-dependence of the bending modulus found in the experiments for nanotubes^10^,^15^. In addition, the Euler-Bernoulli beam model together with the non-ideal BCs was proposed to account for the physics of the observed phenomena. This modified beam model is also found to fit the size-dependent bending modulus of nanotubes measured in the TPB experiments^10^,^15^ very well.

## Simulation Method

**Figure 1:**
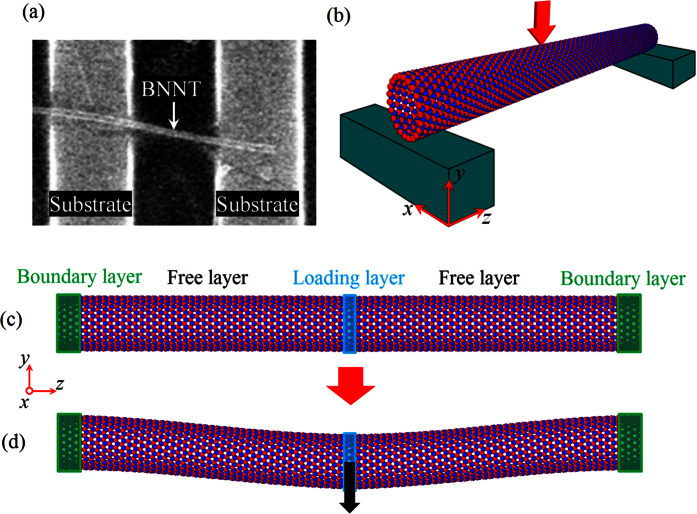
(**a**) Scanning electron microscope image of BNNTs adhered on the patterned substrate. Adapted with permission from ref. 15. Copyright (2012) American Chemical Society. (**b**) Schematic drawing of the AFM-based TPB test of a BNNT. (**c**) MD TPB simulation model of a nanotube. The nanotube is divided into three sections, including boundary layer, free layer and loading layer. (**d**) The deformed shape of during the TPB simulation.

In the present study, we take the BNNT as an example to investigate the effect of the BCs on the bending properties of the tubular nanostructures. To conduct the computational calculations, the entire nanotube was divided into three sections, i.e., (1) the boundary layers at the two ends, which correspond to the portion of the nanotubes attaching to the substrate, (2) the moving layer at the middle of the nanotube, which is used to apply the equivalent transverse displacement loads produced by the AFM tip, and (3) the free layers, which are between the moving and boundary layers. The free layers together with the moving layer are equivalent to the suspended portion of the nanotubes in the AFM-based TPB test. In this study, the classical MD simulations were employed to investigate the bending behaviours of BNNTs. In the present MD simulation studies, the interactions between the boron and nitrogen atoms were described by Tersoff potentials^20^, where the parameters were adopted from^21^ and have been successfully employed to evaluate the mechanical properties of SW BNNTs^21^. Here, the energy-minimized configuration of BNNTs was obtained via the conjugate gradient method. Then MD simulations were performed with the following procedure. First, the BNNT was completely relaxed for a certain period (20 ps was used in this work) to minimize the internal energy and reach an equilibrium state. In doing this, the NPT ensemble (constant number of particles, pressure and temperature) was employed to maintain a constant temperature with the aid of the Nosé-Hoover thermostat algorithm^22^. In addition, the velocity Verlet algorithm with the time step of 0.5 fs was utilized to integrate the Hamiltonian equations of motion determined by Newton’s second law. Second, all or a few atoms at the boundary layers were blocked to simulate different BCs. Third, to launch the TPB test a displacement control methodology was adopted to apply the transverse deflection to the nanotubes, i.e., atoms in the moving layer were moved transversely, while all or a few atoms at the boundary layers were fixed (see Fig. 1c). Last, the moving layer was kept fixed and the system was relaxed for 1 ps so as to allow the BNNT to reach a new equilibrium state.

By repeating the above process, the nanotubes can be bent continuously until the required deflection has been obtained. In the present study, all MD simulations were conducted using a large-scale atomic/molecular massively parallel simulator (LAMMPS)^23^ with a periodic BC along the axial direction and a constant temperature of 1 K. Here, we selected such a low temperature to simulate the elastic bending behaviour of nanotubes because other deformation mechanisms occur rarely at the temperature of 1 K.

## Results and Discussion

Based on the aforementioned MD simulation technique, we will conduct the TPB test for the BNNTs. The influence of the shear effect and the imperfect BCs on the bending modulus of BNNTs will be quantified. The phenomena observed in MD simulations will be further analysed by using the continuum mechanics theories. In addition, based on MD simulations and the continuum mechanics theories we will reveal the correlation between the imperfect BCs and the size-dependent bending modulus of nanotubes reported in existing experiments^10^,^15^.

### Influence of the loading rate on the bending modulus of SW BNNTs

**Figure 2:**
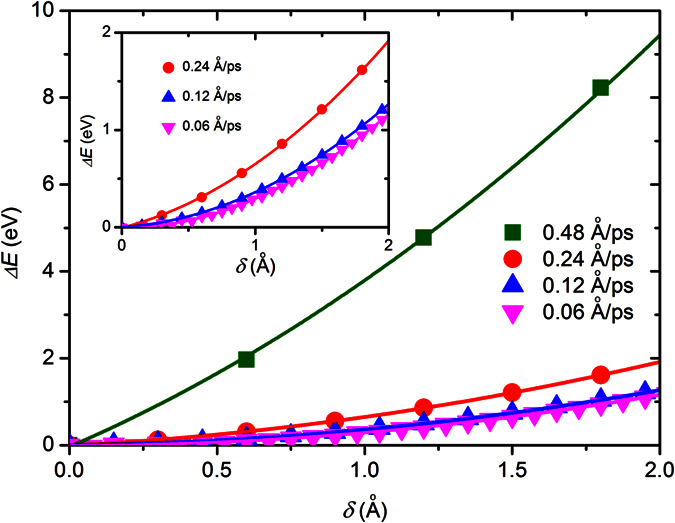
The energy change Δ*E* vs the deflection *δ* of SW BNNTs under different loading rates. Here, the symbols are MD simulation results, while the lines denote the curve fitting to the MD simulation results based on Eq. 2.

To simulate the similar quasi-static loading condition utilized in the experiments^9^,^10^,^11^,^12^,^13^,^14^,^15^, we need to firstly determine the loading rate utilized in the present study. According to some previous studies^24^,^25^,^26^, the mechanical properties of nanotubes strongly depend on the loading rate. Specifically, some recent studies show that when the loading rate is relatively high, the external load will make the CNTs and BNNTs lose their structural integrity^25^,^26^. Thus, it is necessary to study the influence of the loading rate on the bending properties of BNNTs. To this end, based on the technique proposed above we have conducted a series of simulations on a (22, 0) zigzag BNNT (the diameter *D* is ~17.86 Å) with a length *L* about 190 Å. In the simulations all atoms at the boundary layers were fully blocked. Moreover, to study the influence of the loading rate on the bending properties, the atoms at the moving layer of the simulated BNNTs moved transversely with different velocities ranging from 0.06 Å/ps to 0.48 Å/ps. During the TPB simulations we recorded the relationship between the deflection *δ* and the stored strain energy *U* of the BNNTs. In Fig. 2 we plot the *U*-*δ* curves of the BNNTs under different loading rates. We can see from Fig. 2 that when the loading rate is smaller than 0.12 Å/ps *U*-*δ* curves almost overlap with each other, which means that when the loading rate is smaller than 0.12 Å/ps the influence of the loading rate almost can be ignored. Under this situation the loading condition can be equivalently treated as the quasi-static loading. Moreover, we can see from Fig. 2 that in the small deformation range *U* of all simulated nanotubes increases almost quadratically with *δ*. This *U*-*δ* relationship offers a means to calculate the bending modulus of the nanotubes. In order to obtain the bending modulus, a continuum model should be introduced. Generally, a nanotube can be most conveniently modelled as a beam model. Based on the Euler-Bernoulli beam theory the deflection *δ* is linearly proportional to the load *F* and is expressed by^27^ *δ* = *FL*^3^/192*E*_*b*_*I*. Thus, during bending the nanotubes the increase of total energy due to the work done by the applied load is *U* = *Fδ*/2 = 96*E*_*b*_*Iδ*^2^/*L*^3^. This beam model thus leads to the bending modulus of the nanotube as


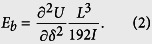


By fitting Eq. 2 to the MD simulation results shown in Fig. 2 we can see that, comparing to the case of the quasi-static loading, a relatively high loading rate will greatly increase the measured bending modulus of nanotubes. For example, we find that the bending modulus of the nanotubes obtained under the loading rate of 0.48 Å/ps is over two times larger than that obtained under the loading rate of 0.12 Å/ps. Therefore, to avoid the influence of loading rate and simulate the similar quasi-static loading condition utilized in the experiments^9^,^10^,^11^,^12^,^13^,^14^,^15^, in the following discussion we choose the loading rate as 0.12 Å/ps.

### Influence of the BCs on the bending modulus of SW BNNTs

**Figure 3:**
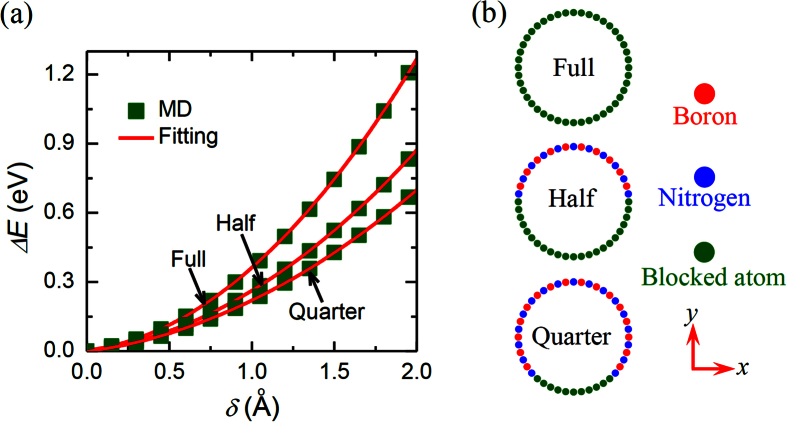
(**a**) The energy change Δ*E* vs the deflection *δ* of SW BNNTs with various BCs as schematically depicted in (**b**).

In this subsection we will start to study the influence of the BCs on the bending properties of SW BNNTs. A similar zigzag BNNT as illustrated in the above subsection was considered here. However, to study the influence the BCs three different BCs were considered for the nanotubes, i.e., all, half or a quarter of the atoms at the boundary layers of the nanotubes were blocked (see Fig. 3). *U*-*δ* curves of the simulated BNNTs were recorded during the TPB test. The recorded *U*-*δ* curves are plotted in Fig. 3 for SW BNNTs with different BCs. We can see from Fig. 3 that during the bending process the strain energy stored in the nanotubes with partially fixed ends is smaller than that with fully fixed ends. Accordingly, we can expect from Eq. 2 that the influence of the imperfect BCs will reduce the bending modulus of nanotubes. Indeed, through fitting Eq. 2 to the MD simulation results depicted in Fig. 3 we find that the bending modulus of the nanotubes with half and a quarter of atoms at the boundary layers being blocked is respectively 35% and 54% smaller than that of the nanotubes with all atoms at the boundary layers being blocked.

**Figure 4:**
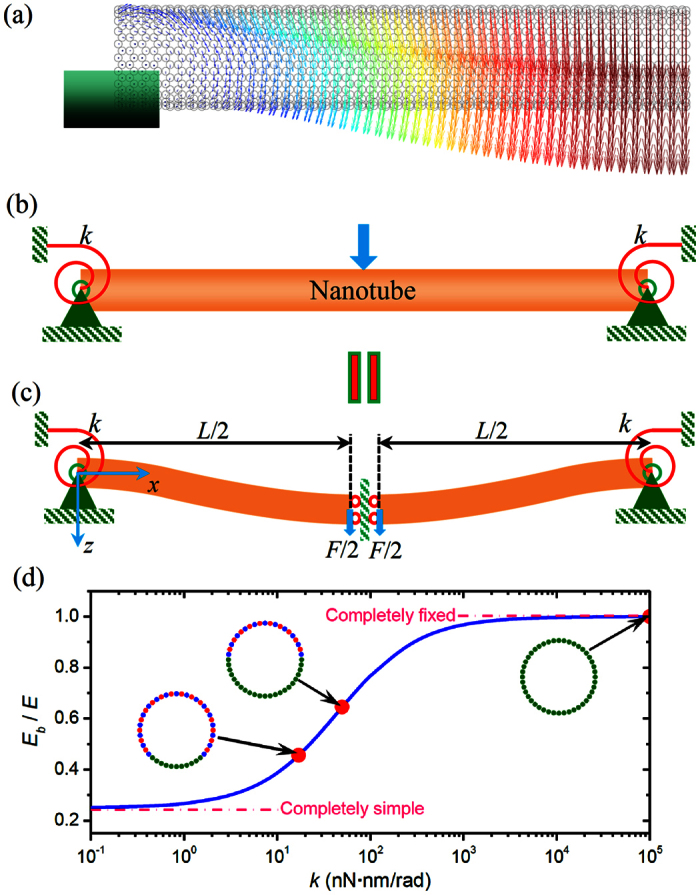
(**a**) Vector plot of atomic displacements of the SW BNNT during the TPB test. (**b**) The continuum mechanics model of a BNNT supported by the torsional spring at the end. (**c**) Schematic of the continuum mechanics model of a BNNT in bending. (**d**) The elastic modulus ratio *E*_*b*_/*E* as a function of the stiffness of the torsional spring *k*. Here, the solid circles correspond to the results measured in Fig. 3a.

To better understand the influence of the imperfect BCs on the bending properties of the nanotubes, in Fig. 4a we show the atomic displacement of the nanotubes with half fixed BCs during the TPB simulation. Considering the fact that the displacement of the nanotubes is symmetric to the midpoint, in the present paper we only show the results of the left half part of the nanotubes. From Fig. 4a we can see that for the nanotubes with half fixed BCs, in the portion attaching to the substrate some atoms also have relative torsional displacements to the blocked atoms. Moreover, during the simulations the atomic stress of the nanotubes with fully and partially fixed ends is shown in Movie 1 and Movie 2, respectively. In the movies the atoms are coloured according to the atomic stress along the length direction. We can see from these movies that in the region nearby the boundaries the atomic stress of the nanotubes with partially fixed ends is much smaller than that with fully fixed ends. Accordingly, in the region nearby the boundaries the moment (proportional to the stress) generated in the nanotubes with partially fixed ends is much smaller that generated in the nanotubes with fully fixed ends. These results suggest that the perfectly fixed ends widely utilized in the previous studies^9^,^10^,^11^,^12^,^13^,^14^,^15^ cannot exactly describe the real BCs of the nanotubes in the TPB test and a general BC should be introduced for nanotubes as far as the beam model is employed. Thus, as shown in Fig. 4b, we assume that the ends of the nanotubes are restricted by a torsional spring with the coefficient of *k* rather than perfectly fixed.

It is known that when a transverse force *F* is applied at the midpoint of a beam as shown in Fig. 4b, its deflection should be symmetric to the midpoint. Then, the deflection of the whole beam can be equivalently represented by its left (or right) half part with the force and BCs as shown by Fig. 4c. Based on the Euler-Bernoulli beam theory, the differential equation of the static deflection of the beam illustrated in Fig. 4c is expressed as


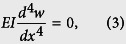


where *E* is Young’s modulus, *w* is the transverse deflection and *x* is the coordinate. The BCs of the beam in Fig. 4c are hinged with a rotational spring at *x* = 0 and free to move laterally with an applied force at *x* = *L*/2, that is


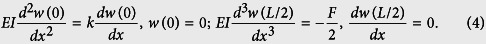


Integrating Eq. 3 four times successively with respect to *x* yields an algebraic equation with four constant coefficients, which generally can be written as


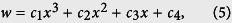


where *c*_1_ − *c*_4_ are the constants of integrations. Using Eq. 4 in Eq. 5 gives


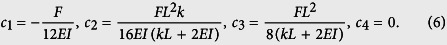


Substituting Eq. 6 into Eq. 5 yields the analytical expression of the beam profile


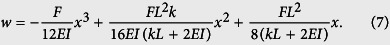


The maximum deflection *δ* of the beam at *x* = *L*/2 is calculated according to Eq. 7 by


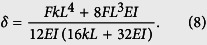


Thus, when the influence of the imperfect BCs is considered, Eq. 1 can be rewritten to give the deflection as


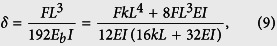


Based on Eq. 9, in Fig. 4d we show the influence of the spring coefficient *k* on the elastic modulus ratio *E*_*b*_/*E*. Here, for the SW nanotubes their second moment of area *I* can be written as *I* = *πD*^3^*t*/8, where *t* is the equivalent wall-thickness of the BNNT and usually assumed to be 3.4 Å^28^. We can see from Fig. 4d that when *k* is relatively large the ratio *E*_*b*_/*E* approaches one, which means that when the torsional spring is relatively large the BCs of the equivalent beam model of the nanotubes can be equivalently treated as the perfectly fixed ends. In this case the bending modulus obtained from the TPB test is equivalent to the Young’s modulus of the nanotube. On the other hand, the ratio *E*_*b*_/*E* is found to decrease as *k* decreases and approaches 0.25 when *k* is relatively small. Actually, when *k* ~ 0 the nanotube should be equivalently regarded as a simply supported beam and, accordingly, the deflection should be^27^ *δ* = *FL*^3^/(48*EI*). Comparing this equation to Eq. 1 we see that in this case the Young’s modulus is four times greater than the bending modulus predicted based on Eq. 1. In other words, when the torsional spring is relatively small, Eq. 1 which was widely utilized in the TPB experiments^9^,^10^,^11^,^12^,^13^,^14^,^15^ will greatly underestimate the Young’s modulus of nanotubes. Moreover, by fitting the present continuum mechanics solution (Eq. 9) to the MD simulation results (shown in Fig. 3), we can obtain the equivalent stiffness of the torsional spring utilized in the continuum mechanics model of the SW BNNTs with partially fixed ends. The results are shown in Fig. 4d, where the equivalent stiffness of the torsional spring is found to decrease as the number of the blocked atoms of the BNNTs decreases.

### Influence of the BCs on the bending modulus of MW BNNTs

**Figure 5:**
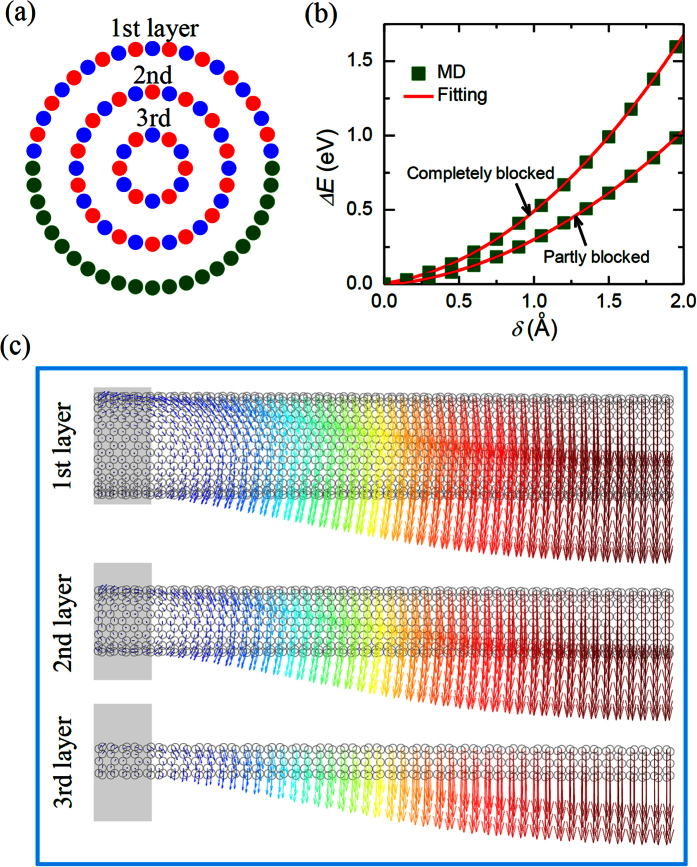
(**a**) Schematic diagram of the imperfect BC of an MW BNNTs. (**b**) The energy change Δ*E* vs the deflection *δ* of MW BNNTs with fully fixed and partially fixed BCs. (**c**) Vector plot of atomic displacements of each layer of the MW BNNTs during the TPB test. Here the gray rectangle shows the boundary region of the BNNTs.

In the above analysis, we have studied the influence of the BCs on the bending behaviours of SW BNNTs. Subsequently, we will investigate how the BCs affect the corresponding bending properties of MW nanotubes. To this end, we considered a (6, 0)@(14, 0)@(22, 0) triple-walled BNNT, where the tube layers are stacked inversely (see refs 29 and 30 for details). In order to model the long-range van der Waals (vdW) interaction for the interlayer interaction, the original Tersoff potential energy proposed above is extended by adding a long-range Lennard-Jones (LJ) 12–6 potential. The LJ parameters employed in the present simulation were adopted from^31^. Similar to the above SW BNNT, here the length *L* of the MW BNNT was taken as 190 Å. It is noted that because the inner layers of MW BNNTs are wrapped by the outermost layer, when an MW BNNT attaches to the substrate, only the atoms at the outermost layer of the MW BNNT can be blocked. Similar to the above studies of the SW BNNTs, to study the influence of the BCs on the bending properties of MW BNNTs, at the two edges of the outermost layer of BNNTs half atoms were blocked (see Fig. 5a). The *U*-*δ* relationship of such MW BNNT obtained in the TPB simulation is plotted in Fig. 5b, where, for the sake of comparison, the results of the MW BNNT with all atoms at the boundary layers being blocked are also presented.

Through fitting Eq. 2 to the MD simulation results depicted in Fig. 5b we find that the bending modulus of the MW BNNTs with half fixed BCs is 40% smaller than those with fully fixed BCs. Although the influence of the imperfect BCs on the bending modulus is qualitatively the same for both SW and MW BNNTs, the influence of the imperfect BCs on the MW BNNTs is stronger than that on the SW BNNTs as the imperfect BCs reduce the bending modulus of the SW BNNTs by only 35%. To shed some light on the observed difference between the SW and MW BNNTs, in Fig. 5c we show the atomic displacement of the MW BNNTs with half fixed BCs during the TPB test. From Fig. 5 we can see that the atomic displacement of the outermost layer of the MW BNNTs is similar to that of their SW counterparts, where some atoms in the portion attaching to the substrate have relative torsional displacements to the blocked atoms. As for the inner layers of the MW BNNTs with half fixed BCs, a rotation displacement is also detected in the boundaries of the second and third layers since the displacements of the atoms in the inner layers of the MW nanotubes are only restricted by the weak vdW forces. It is worth emphasizing that such rotation displacement in the boundaries of the inner layers of the MW nanotubes was ignored in the previous studies^9^,^10^,^11^,^12^,^13^,^14^,^15^, where the boundaries of each layer of the MW nanotubes were tacitly assumed to be completely fixed. Considering the contribution of the additional rotation displacement in the boundaries of the inner layers, it is reasonable to expect that the influence of the imperfect BCs on the bending properties of the MW BNNTs should be more significant than that on the SW BNNTs, which is consistent with our MD simulation results.

### Comparison between the influence of the BCs and the shear deformation

**Figure 6:**
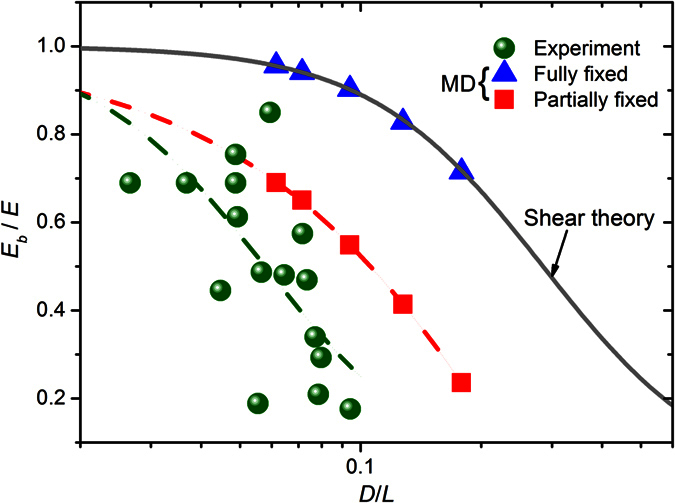
The elastic modulus ratio *E*_*b*_/*E* as a function of the diameter-to-length ratio *D*/*L* of the MW BNNTs with fully fixed and partially fixed BCs. For comparison purpose, the experimental data (solid circles) adapted from^15^ is also shown. Here, the dashed lines are polynomial fits drawn to guide the eye.

As we mentioned in the introduction, a unique size-dependent bending modulus of nanotube was reported in most existing TPB experiments^9^,^10^,^11^,^12^,^13^,^14^,^15^, and the shear effect was widely accepted as a possible explanation for such size-dependent bending modulus. However, those studies were all based on the “curve fitting technique” by initially assuming that the size-dependence phenomenon originates from the shear effect. Thus, a direct measurement of the influence of the transverse shear deformation is still required. Moreover, we can see from above discussion that in addition to the shear effect the imperfect BCs can also greatly influence the bending modulus of nanotubes and thus can be regarded as another possible factor that may induce the size-dependent bending modulus of the nanotubes. To reveal the physics behind this size-dependence phenomenon we need to respectively quantify the influence of these two factors: the transverse shear deformation and the imperfect BCs. Firstly, we will quantify the influence of the transverse shear deformation. According to the Timoshenko beam theory^27^, when the transverse shear deformation is considered Eq. 1 can be rewritten to give the total deflection modulus of a beam with perfectly fixed BCs^9^,^11^, which is shown as follows


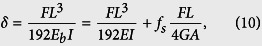


where *f*_*s*_ is a shape factor and equals to a value of 10/9 for a cylindrical beam^9^,^11^, *G* is the shear modulus and *A* is the cross-sectional area. After expanding *I* and *A*, we obtain the equivalent bending modulus of a beam considering the transverse shear deformation as follows


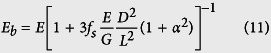


where *α* = *d*/*D* is the ratio of the inner diameter *d* to the outer diameter *D* of nanotubes. The above Timoshenko beam theory (Eqs 10 and 11) converges to the Euler-Bernoulli beam theory when the beam is rigid in shear, i.e., *G* → ∞. In this case, the bending modulus is found to be equal to the Young’s modulus. We can see from Eq. 11 that, in terms of the influence of the geometry of the nanotubes the effect of the transverse shear deformation on the bending modulus of the nanotubes is mainly determined by their diameter-to-length ratio (*D*/*L*). Inspired by this idea, to quantify the influence of the transverse shear deformation on the bending behaviours of nanotubes, using the simulation technique proposed above we calculated the bending modulus of five (6, 0)@(14, 0)@(22, 0) triple-walled BNNTs, whose length is respectively 290 Å, 250 Å, 190 Å, 140 Å and 100 Å (the diameter-to-length ratio is accordingly 0.094, 0.128, 0.179, 0.071 and 0.062). Here, to eliminate the influence of the imperfect BCs all atoms at the boundary layers of the BNNTs were completely blocked. The obtained elastic modulus ratio *E*_*b*_/*E* of the simulated BNNTs is plotted in Fig. 6 (triangles) as a function of *D*/*L*. Here, the Young’s modulus *E* was obtained by fitting Eq. 11 to the obtained MD simulation results. For the sake of comparison, the experimental results reported by Tanur *et al*.^15^ are also presented in Fig. 6. In the experiment conducted by Tanur *et al*.^15^ the MW BNNT suspension was firstly dropped onto clean silica substrates patterned and was allowed to dry. After this, the TPB test technique as we described in the introduction was utilized to measure the bending modulus of the BNNTs. In their TPB test a nanotube was ideally assumed as a homogeneous isotropic beam model^15^. Additionally, the ends of the beam model of the nanotubes were assumed to have perfectly BCs (completely simply supported or fixed). Based on these assumptions the bending modulus of the nanotubes measured in the TPB test was found to decrease with increasing diameter-to-length ratio, which was explained by the shear theory. We can see from Fig. 6 that when atoms at the boundary layers of the BNNTs were completely blocked *E*_*b*_/*E* obtained by MD simulations (triangles) increases with decreasing *D*/*L*, which is qualitatively similar to the experimental observations (circles)^15^. In this case the size-dependent bending modulus obtained in MD simulations is attributed to the shear effect, since through fitting Eq. 11 to the MD simulation results we obtain the shear modulus *G* as 245 GPa, which agrees well with 250 GPa that obtained through the torsion test^32^. On the other hand, in quantity the gap between the present simulation results and the experimental results is huge. For example, when *D*/*L* drops to 0.1 *E*_*b*_/*E* obtained in MD simulations of the BNNTs with fully fixed BCs is 0.9, which means that when *D*/*L* < 0.1 the influence of the transverse shear deformation almost can be ignored due to the fact that when *D*/*L* < 0.1 the shear effect reduces the bending modulus by no more than 10%. However, in the same range of *D*/*L E*_*b*_/*E* of the BNNTs that measured in the experiment^15^ still strongly depends on the geometric size of the BNNTs. The significant difference in quantity between the MD simulation results only considering the shear effect and the experiment results clearly shows that the transverse shear deformation may not be the main reason for the size-dependent bending modulus that observed in existing TPB experiments of BNNTs^15^. Thus, caution must be exercised when the influence of the transverse shear deformation is considered to explain the experimental data. It is noted here that the current conclusion can be extended from the present BNNTs to CNTs. It is known that BNNTs and CNTs have comparable Young’s modulus and shear modulus^32^, thus the influence of the shear effect on the the equivalent bending modulus of CNTs is quantitatively close to that on the BNNTs (see Eq. 11).

Then, to quantify the influence of the imperfect BCs on the size-dependence of the bending modulus, we simulated the same five triple-walled BNNTs with different lengths (or different diameter-to-length ratios). Here, to take the influence of the imperfect BCs into account half atoms at the boundary layers of the outermost layer were blocked (see Fig. 5a). The results of *E*_*b*_/*E* obtained in the simulations are plotted in Fig. 6 (squares) as a function of *D*/*L*. Similar to the influence of the shear effect, from Fig. 6 we see that the influence of the imperfect BCs also lead to the size-dependent bending modulus of the nanotubes, where the bending modulus declines with increasing *D*/*L*. Specifically, the imperfect BCs are found to exert more substantial influence on the bending modulus. For example, when *D*/*L* increases from 0.1 to 0.18 *E*_*b*_/*E* of the nanotube with partially fixed BCs decreases from 0.55 to 0.24, whereas in this process the result of the nanotube with fully fixed BCs is found to decrease from 0.9 to 0.72. It is noted in Fig. 6 that both theoretical predictions are qualitatively similar to the experimental observations. But the theory of the imperfect BCs is found to be even closer to the experiment^15^. The qualitative agreement between the proposed theory and the experiments shows the relevance of the imperfect BCs to the size-dependent bending modulus of nanotubes observed in the TPB experiments^9^,^10^,^11^,^12^,^13^,^14^,^15^.

In the meantime, in Fig. 6 a detectable discrepancy is still observed between the experimental results and the simulation results when the effect of the imperfect BCs is considered. Specifically, comparing with the results (squares) obtained in the present simulations, the bending modulus measured in the experiment^15^ drops more significantly with increasing diameter-to-length ratio. This discrepancy can be possibly attributed to the different geometric sizes of the nanotubes between these two studies. Different to the shear theory (Eq. 11), where the bending modulus of the nanotubes only depend on the diameter-to-length ratio (*D*/*L*) (see Eq. 11), in the theory of imperfect BCs (Eq. 9) the bending modulus show a more complex relationship with the geometric size of the nanotubes. For example, as we will illustrate in the following subsection, when the influence of the imperfect BCs is considered, the bending modulus of the nanotubes with longer length will drop more significantly with increasing *D*/*L*. This result is in coincidence with the results observed in Fig. 6, where the nanotubes tested in the experiment^15^ is longer than those simulated in the present study.

### Relevance to the experimental observations

**Figure 7:**
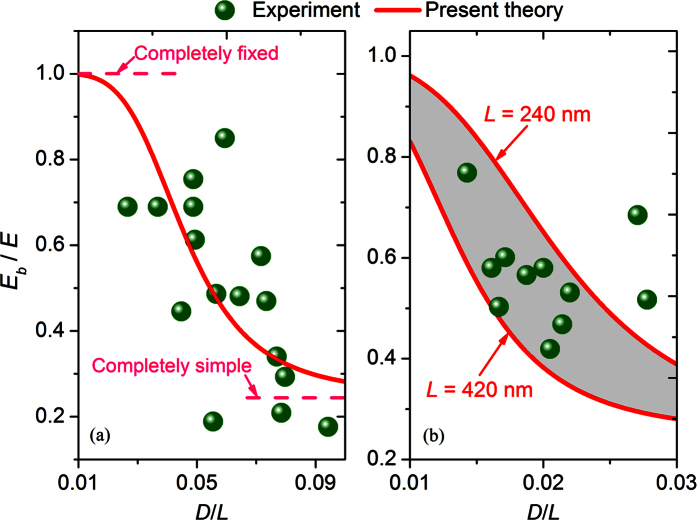
The relevance of the present theory of imperfect BCs to the size-dependent bending modulus of (**a**) BNNTs^15^ and (**b**) CNTs^10^ measured in experiments.

Finally, we will show the correlation between the beam model considering the imperfect BCs (Eq. 9) and the experimental observations. In Fig. 7a and b (circles) we respectively show the experimental results of the elastic modulus ratio *E*_*b*_/*E* of BNNTs^15^ and CNTs^10^ as a function of their diameter-to-length ratio *D*/*L. E*_*b*_/*E* of the BNNTs and the CNTs both apparently decreases with increasing *D*/*L*.

In Fig. 7a we give a curve fitting to the experimental data of MW BNNTs using Eq. 9, which is based on the beam model with the imperfect BCs. Here, the MW BNNT is simply treated as a filled cylinder and thus the second moment of area *I* = *πD*^4^/64. In addition, the length *L* of the BNNTs is 400 nm and approximately the same in all cases^15^; the Young’s modulus *E* of the BNNTs is 1800 GPa as predicted in ref. 15. We can see from Fig. 7a that the present theory of imperfect BCs can well fit the size-dependent bending modulus of BNNTs observed in the experiment^15^ when the spring coefficient *k* is 22 *μ*N · *μ*m/rad. Moreover, we can see from Fig. 7a that when *D*/*L* is relatively small *E*_*b*_/*E* tends to one, which means that the BCs of the nanotubes now can be treated to be completely fixed. On the other hand, when *D*/*L* is relatively large *E*_*b*_/*E* gradually approaches to 0.25, which means that in this case the nanotube tested in the AFM-based TPB test should be considered as a simply supported beam model rather than a fixed beam model.

Similarly, in Fig. 7b we give a curve fitting to the experimental data of MW CNTs^10^ using Eq. 9. Here, the suspended length *L* of the CNTs ranges from 240 nm to 420 nm^10^ and the Young’s modulus *E* of the CNTs is 1400 GPa, which was obtained based on the fitting technique proposed by Tanur *et al*.^15^. We can see from Fig. 7b that Eq. 9 fits the experimental data^10^ well when *k* is 0.18 *μ*N · *μ*m/rad. According to the previous studies^33^, the binding interaction between the CNTs and the silica substrate could be weaker than that between the BNNTs and the substrate. Such smaller binding energy of the interface between the CNTs and the substrate is a possible reason for the smaller *k* detected in the TPB test of CNTs.

In addition, we can see from Fig. 7b that different to the shear theory (Eq. 11), where the bending modulus of the nanotubes only depend on the diameter-to-length ratio (see Eq. 11), in the theory of imperfect BCs (Eq. 9) the bending modulus show a more complex relationship with the geometric size of the nanotubes. For example, for nanotubes with the same diameter-to-length ratio but different lengths, the bending modulus of the nanotubes will increase as the length decreases (see Fig. 7b). Such complex relationship between the bending modulus and geometric size of nanotubes is a possible responsible for the large scattering of the measured experimental data (see Fig. 7b).

## Conclusions

The influence of the imperfect BCs on the bending properties of BNNTs was investigated based on MD simulations. Our results show that the imperfect BCs will reduce the bending modulus of nanotubes. Moreover, the influence of the imperfect BCs on the MW nanotubes is more significant than that on the SW nanotubes. At the same time, to capture the physics behind the MD simulation results a beam model with the general BCs was also proposed.

In addition, similar to the influence of the previously proposed transverse shear effect, the influence of the imperfect BCs will also induce the size-dependence of the bending modulus of nanotubes. However, comparing these two theories quantitatively, we find that the size-dependence phenomenon of the bending modulus induced by the imperfect BCs is more significant than that induced by the shear effect, which suggests that the imperfect BC can be a possible physical origin that leads to the strong size-dependence of the bending modulus found in the TPB experiments for nanotubes^9^,^10^,^11^,^12^,^13^,^14^,^15^. Indeed, the modified beam model proposed in the present study that takes the influence of the imperfect BCs into account is found to fit the experimental data^10^,^15^ very well.

## Additional Information

**How to cite this article**: Zhang, J. Size-dependent bending modulus of nanotubes induced by the imperfect boundary conditions. *Sci. Rep.* **6**, 38974; doi: 10.1038/srep38974 (2016).

**Publisher's note:** Springer Nature remains neutral with regard to jurisdictional claims in published maps and institutional affiliations.

## Supplementary Material

Supplementary Movie1

Supplementary Movie2

Supplementary Information
