# Supplementary material for: Size-dependent bending modulus of nanotubes induced by the imperfect boundary conditions
Source: Sci Rep. 2016 Dec 12;6:38974. doi: 10.1038/srep38974 (PMC5150422; doi:10.1038/srep38974)
Supplement: Supplementary Information [file srep38974-s3.pdf]

Supplementary Information

Size-dependent bending modulus of nanotubes induced by the imperfect boundary conditions

Jin Zhang*

Shenzhen Graduate School, Harbin Institute of Technology, Shenzhen 518055, China

Supplementary Video

Movie 1: The atomic stress of the nanotubes with fully fixed ends.

Movie 2: The atomic stress of the nanotubes with partially fixed ends.

*Corresponding author. E-mail address: zhangjin@hitsz.edu.cn.
